# Effect of nutrition assessment, counselling and support integration on mother-infant nutritional status, practices and health in Tororo and Butaleja districts, Uganda: A comparative non-equivalent quasi-experimental study

**DOI:** 10.1186/s41043-024-00559-7

**Published:** 2024-06-12

**Authors:** Samalie Namukose, Gakenia Wamuyu Maina, Suzanne N Kiwanuka, Fredrick Edward Makumbi

**Affiliations:** 1 Department of Health Policy Planning and Management, School of Public Health, College of Health Sciences, Makerere University, Kampala, Uganda; 2 Department of Community Health and Behavioural Sciences, School of Public Health, College of Health Sciences, Makerere University, Kampala, Uganda; 3 Department of Epidemiology and Biostatistics, School of Public Health, College of Health Sciences, Makerere University, Kampala, Uganda

**Keywords:** Effect, Nutrition assessment counselling support, Health, Nutrition outcomes, Mothers, Infants, Quasi-experimental

## Abstract

**Background:**

Malnutrition remains a health challenge for women aged 15 to 49 years and their infants. While Nutrition Assessment Counselling and Support (NACS) is considered a promising strategy, evidence of its effectiveness remains scanty. This study assessed the effect of the comprehensive NACS package on the mother-infant practices, health and nutrition outcomes in two districts in Eastern Uganda.

**Methods:**

A comparative non-equivalent quasi-experimental design was employed with two groups; Comprehensive NACS (Tororo) and Routine NACS (Butaleja). Pregnant mothers were enrolled spanning various trimesters and followed through the antenatal periods and post-delivery to monitor their health and nutrition status. Infants were followed for feeding practices, health and nutritional status at birth and weeks 6, 10, 14 and at months 6, 9 and 12 post-delivery. Propensity score matching ensured study group comparability. The NACS effect was estimated by nearest neighbour matching and the logistic regression methods. Statistical analysis utilised STATA version 15 and R version 4.1.1.

**Results:**

A total of 666/784 (85%) with complete data were analysed (routine: 412, comprehensive: 254). Both groups were comparable by mothers’ age, Mid Upper Arm Circumference, prior antenatal visits, meal frequency, micronutrient supplementation and instances of maternal headache, depression and diarrhoea. However, differences existed in gestation age, income, family size, education and other living conditions. Comprehensive NACS infants exhibited higher infant birth weights, weight-for-age z-scores at the 3rd -6th visits (*p* < 0.001), length-for-age z scores at the 4th -7th visits (*p* < 0.001) and weight-for-length z-scores at the 3rd − 5th (p < = 0.001) visits. Despite fewer episodes of diarrhoea and fever, upper respiration infections were higher.

**Conclusions:**

The comprehensive NACS demonstrated improved mother-infant nutritional and other health outcomes suggesting the need for integrated and holistic care for better maternal, infant and child health.

## Introduction

Maternal and infant malnutrition is a significant global health concern with significant implications for the overall health and well-being of both mothers and their infants. The Global Nutrition Report of 2022 [1] indicated that 29.9% of women of reproductive age suffer from anaemia, 9.1% are underweight and 14.6% of newborns have low birth weights. Additionally, 22%, 6.7% and 5.7% of children under 5 years were stunted, wasted and overweight respectively. In the same report, Sub-saharan Africa was noted to contribute to the highest burden of malnutrition with 32.6% of children under 5 years stunted, 5.2% wasted and 4% overweight while anaemia among the women of reproductive age was 31.9%. According to the Uganda Demographic Health Survey (UDHS) of 2023 [2], the prevalence of stunting among children under 5 years was 26% while underweight and wasting were 9.7% and 3.2% respectively. Additionally, anaemia affects 32% of women of reproductive age [3]. These surveys and reports indicate the persistent challenge of malnutrition among the women of reproductive health and children calling for urgent need for intervention and improvement.

Maternal nutrition greatly impacts maternal and infant health emphasising the importance of interventions before, during and after pregnancy [4–8]. Well-nourished and healthy mothers are more likely to give birth to healthy babies, experience a healthy pregnancy and are less likely to experience life-threatening complications during pregnancy [9, 10].

Multi-sectoral approaches have shown promising results in improving maternal and infant health with comprehensive interventions like breastfeeding promotion, education and counselling, maternal mental health, women empowerment, family planning, water, hygiene and sanitation, and agricultural interventions yielding positive results in reducing stunting rates [11]. Notable studies by Olutayo et al. [12], Nadia et al. [13], and Bhutta et al. [14] emphasize the importance of these holistic interventions in reducing stunting. However, there are debates regarding the use of stunting as a primary indicator of intervention success, with perspectives advocating for the population well well-being [15].

Nutrition Counselling and education during pregnancy have shown positive effects on maternal-infant nutrition practices, health and nutritional outcomes [16–18], yet challenges remain, including the quality and delivery of counselling [19, 20]. More research is needed to bridge this gap and provide a clearer understanding of the effect of delivery of a comprehensive package including counselling on the mother-infant health and nutrition outcomes.

Providing high-quality health services, including preventive care, early diagnosis and treatment of medical conditions is crucial for improving women’s health. The World Health Organisation (WHO) and the Ministry of Health, Uganda recommend a comprehensive package of nutrition interventions to pregnant women for a positive outcome, including counselling on healthy eating and physical activity, guidance on infant and young child feeding, nutrition education on energy and protein intake, and daily iron and folic acid supplements. The package also includes energy and protein dietary supplements and high-protein supplements for the undernourished populations [21, 22].

Even before the release of the WHO guidelines in 2020, the Ministry of Health in Uganda had been implementing the NACS initiative, aiming to integrate nutrition into the health system and consequently improving the health and nutrition practices and outcomes of the beneficiaries. The NACS intervention package was tailored to the specific nutrition needs of the clients and was in line with WHO’s recommendations on maternal nutrition care [23].

While existing literature has assessed the impact of vertical maternal interventions on the health and nutritional status of mothers and infants, there is a limited body of research on the effect of broad integrated interventions, such as NACS on the health and nutrition outcomes of beneficiaries. This study, therefore, sought to assess the effect of the comprehensive NACS package on the health and nutrition practices and status of mothers and their infants in Tororo and Butaleja districts in Eastern Uganda. We tested the hypothesis that there was no difference in the maternal-infant health, nutrition practices and outcomes between the facilities which integrated comprehensive NACS, versus those with routine NACS. The findings of this study contribute to the growing body of evidence on the effectiveness of broadly integrated interventions on the health and nutrition outcomes of the beneficiaries and provide insights and recommendations for scaling up the NACS approach.

### Pathways on the effect of NACS integration in the health system on maternal and infant health, nutrition practices, and outcomes

**Fig. 1:**
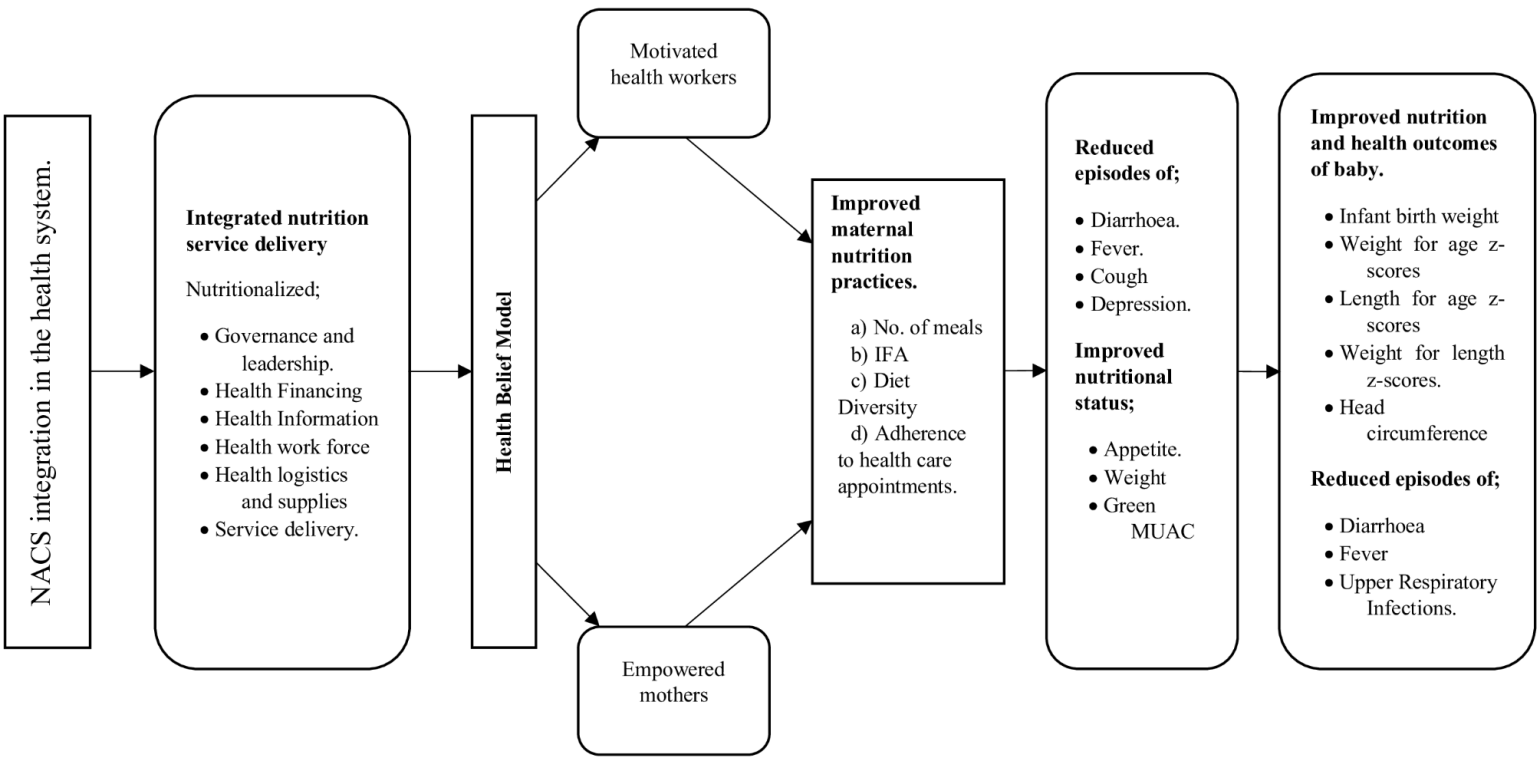
Pathways on the effect of NACS integration in the health system on maternal and infant health nutrition practices and outcomes adapted from the chronic care model [23]

The integration of comprehensive NACS into the health system aims to create an integrated nutrition service delivery system, fostering a productive interaction between the service providers and mothers. Based on the health belief model, empowering mothers with with knowledge and skills by service providers is expected to result in optimal nutrition practices, subsequently improving maternal-infant health and nutrition outcomes as illustrated in Fig. 1.

## Methods

### Study design

The study used a comparative non-equivalent quasi-experimental design with two groups; comprehensive NACS integration compared to routine NACS integration.

### Study setting and population

The study involved pregnant and lactating mothers, along with their respective infants. The two hospitals selected for the study were Tororo Hospital as the comprehensive NACS and Busolwe Hospital as the routine NACS. The hospitals were similar by level of facility, ownership, funding, staffing norms, services provided and client load. Pregnant mothers in various trimesters were enrolled and their health and nutrition status was monitored at the antenatal visits and post-delivery. Only women accessing antenatal care and residing in Tororo and Butaleja districts were included. During post-delivery, infants were monitored for their feeding practices, health and nutrition status till 12 months.

Mothers and infants were followed through the scheduled visits at their respective health service points, which included, antenatal, labour suite/maternity, postnatal, children wards, young child and ART clinics.

#### Comprehensive NACS versus routine service delivery

The comprehensive NACS package targeted both health workers and mothers with their infants. A few health workers were selected from antenatal clinics, maternity, postnatal clinics, and young child and HIV clinics to undergo a five-day training program for each course on NACS and Health Management Information Systems (HMIS). Anthropometric equipment, policy guidelines, job aides, information, education and communication materials were provided to facilitate service delivery. As part of the routine activities, the health workers employed quality improvement approaches to address gaps in nutrition service delivery. They linked mothers and their infants to community support structures to ensure ongoing nutrition care and support. Additionally, they monitored and reported on nutrition interventions while actively collaborating with key stakeholders and the district health management team for sustained support. Continuous mentoring and supervision were provided to ensure quality service provision.

To the mothers and their infants, the package included: nutrition assessment and categorization of the nutritional status; health and nutrition education on a diversified diet, recommended antenatal clinic visits, iron/folic acid supplementation, water hygiene, and sanitation education; maternal-infant nutrition counselling; provision of therapeutic feeds to identified malnourished cases; active follow-up of mother-baby pairs to ensure they receive the necessary nutrition services.

#### Routine service delivery

In the routine NACS setting, some elements of NACS were integrated into the regular health care services provided such as growth monitoring and promotion for children, and iron/folic acid supplementation. To ensure comparability, staff at both study settings were trained in NACS and HMIS for nutrition. They were provided with information, education and communication materials to enhance their capacity in nutrition education and counselling. Subsequently, the staff carried on with their services as usual. The nutrition counselling placed a strong emphasis on promoting the consumption of locally available foods for the management of malnutrition [24]. 

We determined the level of exposure to comprehensive versus routine NACS by closely supervising the data collection process and enhancing documentation of both the services rendered and the frequency with which the respondents accessed these services.

### Sampling

The study employed a purposive sampling approach, enrolling subjects who had given their consent continuously until the desired sample size was attained. In both study settings, the antenatal care clinic served as the entry point and the ANC register as the sampling frame. The enrolment of the study participants took 8 months starting from 23rd October 2018 to 2nd May 2019. The mothers were followed up till they gave birth, and the mother-infant pairs were followed up for 12 months. Data collection commenced on 23rd October and was concluded on 2nd April 2021.

### Sample size calculation

The sample size was calculated using the formular by V. Kasiulevicius et al. [25] based on infant underweight as an outcome variable.

$$\text{n}=\frac{\left[{{\text{z}}_{{\upalpha }}}_{\sqrt{\left(1+\frac{1}{\text{m}}\right) \stackrel{-}{\text{p}}(1- \stackrel{-}{\text{p}})} + {\text{z}}_{{\upbeta }}\sqrt{\frac{\text{p}\text{o}\left(1-\text{p}0\right)}{\text{m}}+ \text{p}1(1-\text{p}1)}}\right]2}{{(\text{p}0-\text{p}1)}^{2}}$$

Where $$\stackrel{-}{\text{p}}=\frac{\text{p}1+\text{m}\text{p}0}{\text{m}+1}$$

Where,

P_0_ was the probability of underweight infants in the control group – 0.113.

P_1_ was the probability of underweight infants in the intervention group – 0.07.

P_0_ was based on the Uganda Demographic Health Survey 2011 burden of malnutrition in the eastern region while P1 was an estimated reduction in underweight with the intervention.

If α (alpha) = 0.05 then z_α_ = 1.96.

If β (beta) = 0.80, then z_β_ = 0.845.

m was the number of control subjects per experimental subject = 2.

$$\stackrel{-}{\text{p}}$$ = 0.0987

*n* = 652 with the inclusion of 20% loss to follow-up of mother-baby pairs. A sample size of 652 (217 in the intervention group and 435 in the control group) was estimated to detect a 4.3% reduction in the underweight infants at 80% power and 5% level of significance.

### Data collection

The questionnaire was adapted from the questionnaires utilized in the Uganda Demographic Health Surveys to collect data on the mother-infant variables. We pre-tested this tool among the mothers and their feedback was used to refine it. The tool was designed in Excel to facilitate tracking of the mother-baby variables for their scheduled visits.

Our research assistants underwent training on the data collection tool and data capture methods at a minimum of four points: recruitment/baseline, antenatal clinic visits, delivery and postnatal care clinic, and immunization scheduled visits. We encouraged mothers to deliver at the health facility where they were provided with a package of both routine and comprehensive package of services. To ensure data quality, we conducted regular supervision and spot checks.

At baseline/recruitment, we collected data on various aspects, including socio-economic and demographic characteristics, maternal health and nutrition practices, and maternal nutritional status. Throughout each antenatal visit, we monitored the mothers’ anthropometric data, health status and nutrition practices. Following delivery, we collected data on the infant’s anthropometric measurements such as birth weight, length, and head circumference as well as details about their feeding practices. Subsequently, we continued to track the infants’ anthropometric data, health and nutrition practices and status during the scheduled immunization visits.

Anthropometric data and feeding practices for both the mother and her infant were collected using standard procedures. The mother’s weight was taken to the nearest 0.1gm using a digital Uniscale. The infant’s weight was measured to the nearest 0.1 gm using the neonatal weighing scales at birth and after that a digital uniscale. Infant length was measured to the nearest 0.1 cm using an infantometer at birth and a height board for the subsequent visits.

We measured the head circumference and Mid Upper Arm Circumference (MUAC) of infants using specialized tapes, with measurements recorded to the nearest 0.1 cm. MUAC was measured for infants above 6 months and mothers. Additionally, we conducted health assessments for mothers, including evaluation for illnesses such as headaches, depression, diarrhoea, fever and cough. For infants, we assessed episodes of diarrhoea, fever and Upper Respiratory infections.

In total, there were 15 scheduled appointments from the time of the mother’s enrolment until the baby was 12 months of age. Mothers were encouraged to continue attending health facilities for continuous health care as well as participating in informative health and nutrition education sessions.

### Data management

We used Excel for data capture, STATA version 15 for data, cleaning and performing bivariate tests on all confounding background variables for both study settings. The variables included; weeks of gestation, age of the mothers, mothers’ education, marital status, mothers’ occupation, mothers’ income, spouses’ income, spouses’ education level, previous ANC visits, distance to the health facility, type of transport used, total numbers of children, number of children alive, number of family members, number of children under 5 years, fuel for cooking, water source and faecal matter disposal. We cleaned data by synchronising the variable codes for the two data sets, checked for missing data, and excluded the variables with insignificant data.

We characterised variables as continuous, binary, and categorical and generated new variables. We checked the data set for normal distribution for the continuous variables. Descriptive analysis was conducted to compare mothers’ background characteristics in the 2 study arms. Continuous variables were compared using a 2 sample t-test while the categorical variables were compared using the chi-square test.

### Data analysis

Because these groups were not randomly assigned and this was a non-equivalent quasi-experimental study, we conducted propensity score matching to minimise potential imbalance and also create reasonably comparable groups, before assessing the effectiveness of the NACS intervention.

By creating more comparable intervention and control groups, propensity score matching resulted in a more precise estimate of intervention effects and reduced confounders. On the other hand, matching reduced the sample size, because not all individuals found suitable matches resulting in a loss of statistical power and precision [26–28].

The propensity score matching process involved; defining the intervention (comprehensive NACS) versus control (routine NACS) groups, identification of the variables before administration of the intervention, estimating the propensity scores, checking the initial balance of the variables for both groups using mean differences, using the nearest-neighbour matching method to pair individuals in intervention and control group based on their propensity scores, assessing the quality of the matches, and after that estimating the effect of comprehensive NACS on maternal-infant practices, health and nutritional status [28].

**Fig. 2:**
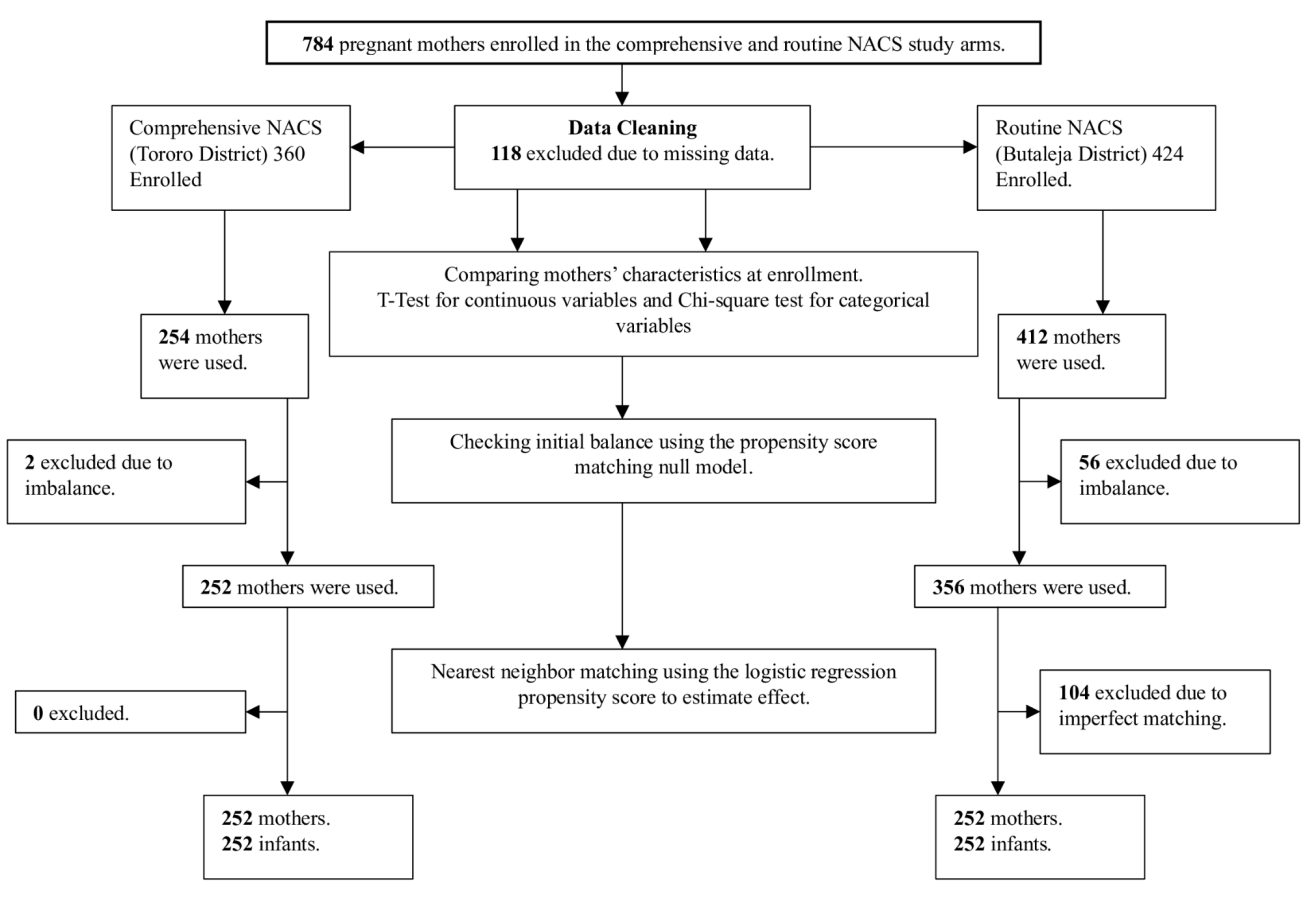
Enrolment and data analysis flow chart

Using the R software version 4.1.1, the NACS effect on maternal-infant nutrition practices, health and nutritional status was estimated by comparing various methods such as nearest neighbour, null and full matching methods. The nearest neighbour matching using the logistic regression propensity score model provided the best balance compared to other matching methods such as; full matching using a probit regression propensity score, nearest neighbour matching using a probit regression propensity score, null probit, full matching using a logistic regression propensity score as determined by the lower standardised mean difference statistics. The enrolment and data analysis flow chart is illustrated in Fig. 2.

## Results

A total of 784 mothers were enrolled in the study; 423 from the Routine NACS setting and 361 from the Comprehensive NACS setting. One hundred (118) mothers were excluded from the analysis due to missing data while 666 mothers were considered in the final analysis, the majority from the routine (412) compared to comprehensive (254) NACS groups.

The mothers’ characteristics at enrollment were compared in the two study arms and the results are shown in Tables 1 and 2. The findings indicated no significant difference between the mother’s age (*p* = 0.466), prior antenatal visits for this pregnancy (*p* = 0.316), number of family members (*p* = 0.007) and weeks of gestation (*p* = 0.023) between the two groups at enrollment. However, there was a significant difference in, mothers’ and spouses’ income (*p* = 0.000), number of children (*p* = 0.000), number of children alive (*p* = 0.000), number of children < 5 years (*p* = 0.001), distance to health facility (*p* < 0.001), mothers’ education (*p* < 0.001), marital status (*p* < 0.001), mothers’ occupation (*p* < 0.001), spouses’ education (*p* < 0.001), type of transport (*p* < 0.001), cooking method (*p* < 0.001), water source (*p* < 0.001), and faecal matter disposal (*p* < 0.001).

**Table 1:** Mothers’ characteristics at enrolment in the routine versus comprehensive study arms for continuous variables

Variable name (Comprehensive NACS = 254, Routine NACS = 412)	t-test	*p*-value
Mothers’ age	-0.730	0.466
Weeks of gestation	2.277	0.023
Mothers’ income	-4.682	0.000
Spouse income	-4.997	0.000
Prio antennal visits for this pregnancy	1.003	0.316
Number of children	11.400	0.000
Number of children alive	5.725	0.000
Number of family members	2.669	0.007
Number of children less than 5 years	6.842	0.000

**Table 2:** Mothers’ characteristics at enrolment in the routine versus comprehensive NACS study arms for categorical variables before propensity score matching

Variable name	Routine NACS N = 412	Comprehensive NACS N = 254	Chi-square Test (p-Value)
Distance to health facility			
< 5 Km	96.10%	60.60%	< 0.001
> 5 km	3.90%	39.40%	
**Mothers Education**			
No Education	5.80%	1.20%	< 0.001
Primary	62.90%	44.90%	
Secondary	26.90%	40.60%	
Higher	4.40%	13.40%	
**Marital Status**			
Married	94.40%	99.60%	< 0.001
Not married	5.60%	0.40%	
**Mothers occupation**			
Formal	6.80%	18.10%	< 0.001
Informal	93.20%	81.90%	
**Spouse’s education**			
No Education	4.20%	1.20%	< 0.001
Primary	49.30%	25.60%	
Secondary	38.70%	55.10%	
Higher	7.80%	18.10%	
**Type of transport**			
Motorised	50.70%	92.10%	
Walking	49.30%	7.90%	< 0.001
**Cooking method**			
Firewood	79.90%	52.80%	< 0.001
Charcoal	19.90%	46.10%	
Gas	0.20%	1.20%	
**Water source**			
Well	0.20%	16.10%	< 0.001
Borehole	96.80%	44.10%	
Tap water	2.90%	39.80%	
**Faecal matter Disposal**			
Latrine	99.30%	89.00%	< 0.001
Toilet	0.70%	11.00%	

Propensity score matching was used to create comparability between the study groups. The Null model was used to check the initial imbalance in the two groups that the matching methods eliminated step-wise. Table 3 shows severe imbalances as reflected by the standard mean differences computed by the R software. All values close to zero in the standard mean differences reflected better matches while those away from zero reflected severe imbalances. The variable ‘number of children’ had the highest difference (-4.7263) indicating severe imbalance while the variable ‘nutrition status by MUAC’ had the lowest difference (0.0019) indicating that mothers in both groups had comparable nutritional status, which conclusions resonate with existing literature [29].

**Table 3:** Propensity score matching null model for checking initial imbalance between the comprehensive and routine NACS study arms

Variables	Means Treated	Means Control	Mean difference
Distance	0.8513	0.1053	3.1896
**Mother age**			
15–24	0.4762	0.514	-0.0758
25–49	0.5238	0.4860	0.0758
**Mothers’ education**			
1. No Education	0.0119	0.0562	-0.4082
2. Primary	0.4444	0.6433	-0.4001
3. Secondary	0.4087	0.2556	0.3115
4. Higher	0.1349	0.0449	0.2634
**Marital status**			
1. Married	0.996	0.9438	0.8305
2. Not Married	0.0040	0.0562	-0.8305
**Mothers’ occupation**			
1. Formal	0.1825	0.0674	0.298
2. Informal	0.8175	0.9326	-0.298
**Spouse education**			
1. No Education	0.0119	0.0365	-0.2269
2. Primary	0.2579	0.5056	-0.5661
3. Secondary	0.5476	0.382	0.3327
4. Higher	0.1825	0.0758	0.2762
**Prior ANC visits**	2.0397	2.1011	-0.0626
**Distance to hospital**			
1. Less than 5km	0.6111	0.9635	-0.7228
2. ≥ 5km	0.3889	0.0365	0.7228
**Type of transport**			
**1**. Walking	0.0794	0.5028	-1.5665
2. Motorized	0.9206	0.4972	1.5665
**No. of children**	1.0198	2.6236	-4.7263
**No. of children alive**	1.5437	2.4129	-0.5786
**No of chn < 5years**	0.754	1.2809	-0.6142
**Type of fuel used**			
1. Firewood	0.5278	0.7978	-0.5408
2. Charcoal	0.4603	0.2022	0.5178
3. Gas	0.0119	0.0000	0.1098
**Water source**			
1. Well	0.1627	0.0028	0.4332
2. Borehole	0.4405	0.9691	-1.0648
3. Tap Water	0.3968	0.0281	0.7537
**Faecal matter disposal**			
1. Latrine	0.8889	0.9916	-0.3267
2. Toilet	0.1111	0.0084	0.3267
**Mother weight (kg) 1st visit**	64.2496	60.7542	0.3124
**Nutritional status by MUAC**			
**1.** Normal (Green)	0.9722	0.9719	0.0019
2. Malnourished (Yellow/Red)	0.0278	0.0281	0.0019
**No. of meals**	3.0992	2.8792	0.294
**Iron folic acid supplement**			
No	0.0238	0.0028	0.1377
Yes	0.9762	0.9972	-0.1377
**History of headache**			
No	1	0.8034	0.6465
Yes	0.0000	0.1966	-0.6465
**History of depression**			
No	1	0.9803	0.1851
Ye**s**	0	0.0197	-0.1851
**History of diarrhoea**			
No	1.0000 0.0000	0.9888	0.1393
Yes		0.0112	0.1393
**History of fever**			
No	0.996	0.9298	1.0539
Yes	0.0040	0.0702	-1.0539
**History of cough**			
No	1	0.9522	0.2927
Yes	0	0.0478	-0.2927

### Effect NACS integration on the mothers-infant health, nutrition practices and status

The study assessed the effect of NACS integration on maternal-infant nutrition practices as well as its effects on health and nutritional status. This assessment employed the nearest neighbour matching method along with a logistic regression propensity score model and the results are shown in Table 4.

**Table 4:** The effect of NACS integration on the mother-infant nutrition practices, health and nutrition status using nearest neighbour matching logistic regression propensity score model

Variable name	Contrast		Estimate	SE	P value	CI
	1- (Comp NACS)	0- (Routine NACS)				
**Mother variables** N = 252 N = 252						
**Meal frequency at 2nd visit**	1	0	0.008	0.07	0.911	-0.13, -0.146
**Diet Diversity Score**						
2nd visit	1	0	-3.11	0.263	< 0.001	-3.630, − 2.600
3rd visit	1	0	-2.98	0.213	< 0.001	-3.400, -2.560
4th visit	1	0	-2.69	0.149	< 0.001	2.990, -2.400
**Iron/ folic acid supplementation**						
2nd visit	1	0	-0.029	0.01	0.006	-0.049, -0.008
3rd visit	1	0	-0.024	0.01	0.012	-0.043, -0.005
4th visit	1	0	-0.035	0.015	0.017	-0.064, -0.006
**Weight at**						
2nd visit	1	0	1.04	0.272	< 0.001	0.507–1.570
3rd visit	1	0	2.69	0.422	< 0.001	1.860–3.520
4th visit	1	0	5.86	1.99	0.003	1.950–9.760
**Nut.status by (MUAC**)						
2nd visit	1	0	0.038	0.018	0.032	0.003–0.072
3rd visit	1	0	0.026	0.009	0.047	0.008–0.044
4th visit	1	0	0.025	0.015	0.091	0.004–0.054
**History of headache**						
2nd visit	1	0	0.02	0.009	0.02	0.003–0.037
3rd visit	1	0	0.044	0.013	< 0.001	0.020–0.069
4th visit	1	0	0.078	0.022	< 0.001	0.034–0.121
**History of depression**						
2nd visit	1	0	0.013	0.007	0.07	-0.001, -0.026
3rd Visit	1	0	0.008	0.005	0.16	-0.003, -0.018
**Diarrhoea at 3rd visit**	1	0	0.018	0.008	0.03	0.002–0.034
**History of fever**						
2nd visit	1	0	0.02	0.009	0.019	0.003–0.037
3rd visit	1	0	0.049	0.014	< 0.001	0.023–0.076
4th visit	1	0	0.03	0.013	0.021	0.005–0.055
**Infant variables**						
**Infant birth weight**	1	0	0.191	0.1	0.056	-0.005, -0.387
**Infant weight at**						
3rd visit	1	0	1.05	0.111	< 0.001	0.835 - 1.270
4th Visit	1	0	1.13	0.106	< 0.001	0.923–1.340
5th visit	1	0	1.93	0.107	< 0.001	1.720 - 2.140
**Infant head cirm**						
3rd visit	1	0	3.9	0.248	< 0.001	3.410 - 4.380
4th visit	1	0	3.9	0.248	< 0.001	3.410–4.380
5th visit	1	0	0.02	0.009	0.019	0.003–0.037
**Wt for Age Z-scores**						
3rd visit	1	0	1.8	0.233	< 0.001	1.350–2.260
4th visit	1	0	1.7	0.208	< 0.001	1.290–2.110
5th visit	1	0	2.73	0.202	< 0.001	2.330–3.130
6th visit	1	0	0.265	0.159	0.096	-0.047, -0.577
**Length for Age Z-scores**						
4th visit	1	0	0.634	0.246	0.01	0.151–1.120
5th Visit	1	0	0.761	0.222	< 0.001	0.326–1.200
6th visit	1	0	3.99	0.281	< 0.001	3.440–4.540
7th visit	1	0	4.63	1.14	< 0.001	2.470–6.780
**Wt. for Length Z-scores at**						
3rd visit	1	0	6.25	0.475	< 0.001	5.320–7.180
4th visit	1	0	1.46	0.356	< 0.001	0.763–2.160
5th visit	1	0	2.77	0.342	< 0.001	2.100–3.440
**History of Upper Respiratory Infection**						
5th visit	1	0	0.043	0.013	< 0.001	0.018–0.069
6th visit	1	0	0.155	0.023	< 0.001	0.110–0.199
7th visit	1	0	0.294	0.029	< 0.001	0.237–0.350
8th visit	1	0	0.315	0.029	< 0.001	0.258–0.372
**History of infant diarrhoea**						
5th visit	1	0	-0.168	0.08	0.036	-0.324, -0.011
6th visit	1	0	-0.146	0.08	0.067	-0.302- 0.010
7th visit	1	0	-0.033	0.049	0.506	-0.130- 0.064
8th visit	1	0	-0.126	0.067	0.061	-0.130- 0.064
**History of infant fever**						
1st visit	1	0	-0.054	0.03	0.072	-0.113- 0.005
3rd visit	1	0	-0.005	0.012	0.694	-0.029- 0.019
4th visit	1	0	-0.409	0.086	< 0.001	-0.576, -0.241
5th Visit	1	0	-0.111	0.076	0.145	-0.260- 0.038
6th visit	1	0	-0.023	0.062	0.712	-0.144–0.099
7th visit	1	0	0.028	0.05	0.569	-0.070–0.126
8th visit	1	0	-0.027	0.06	0.657	-0.143- 0.090

Mothers in both groups were similar in terms of; meal frequency (*p* = 0.911), iron/folic acid supplementation at the 2nd -4th visits (β <= -0.035), maternal nutritional status by MUAC at the 2nd – 4th visits (β<= 0.038). Additionally, there was no significant difference in maternal instances of; headache at the 2nd -4th visits (β<=0.078), depression at the 2nd -3rd visit (β<=0.013) and diarrhoea at the 2nd -4th (β<=0.049) visits.

Whereas mothers in the routine NACS group had a significantly higher diversity score at the 2nd − 4th visits (*p* < 0.001), the comprehensive group had higher weights at the 2nd − 4th (p < = 0.003) visits. The difference in weights increased with the number of visits right from the time of mothers’ enrolment.

Compared to routine, infants born to mothers in the comprehensive group had significantly higher; birth weights at a 10% level of significance (*p* = 0.056, CI -0.005–0.387), weight-for-age- age at the 3rd -6th visits (*p* < 0.001) with 20% reduction in underweight on average per visit. Furthermore, their length-for-age was significantly higher at the 4th -7th visits (*p* < 0.001). The difference widened with the increasing number of visits. Similarly, the weight-for-length of the comprehensive NACS group was significantly much higher at the 3rd -5th visits (*p* < 0.001). The difference remained constant throughout the subsequent visits.

Unlike the routine group, infants in the comprehensive NACS group had significantly higher episodes of upper respiratory infections at the 5th -8th (*p* < 0.001, β<=0.315). On the other hand, the routine NACS infants experienced significantly higher episodes of; diarrhoea at the 5th -8th visits (p < = 0.061) and fever at the 1st (*p* = 0.072, β=-0.054) and 4th visits (*p* < 0.001, β=-0.409).

## Discussion

The study aimed to assess the effect of NACS integration on mother-infant nutrition practices, health and nutritional status. The findings provide insights into the potential benefits of nutrition integration on the health system on the wellbeing of the mothers and their infants. The key findings in light of existing evidence and their implication are discussed.

The study found no significant difference in meal frequency among the mothers in both study groups, suggesting similar dietary habits. Compared to the comprehensive group, mothers in the routine NACS setting had significantly higher diversity scores on all visits. This disparity may be attributed to the close proximity to the rural settings offering more natural and diverse food choices. Maternal nutritional status by MUAC estimates exhibited no significant differences across the visits in the two study settings. In contrast to the routine group, mothers in the comprehensive displayed significantly higher weights at the 2nd- 4th visits. This implied that the integration of comprehensive NACS had a positive progressive impact on maternal weight gain from the time of enrolment. The findings suggest potential program implications and highlight the need to consider the environmental context when implementing nutrition programs. Future nutrition interventions should therefore be tailored to the specific needs and context of the target population. Furthermore, the study re-enforces, the existing body of evidence indicating that maternal-focused interventions particularly those with a multi-sectoral nature contribute to improved maternal diet diversity, micronutrient intake and overall nutritional status [30–32].

Additionally, there were minimal difference in iron/folic acid supplementation between the two groups at the 2nd − 4th visits implying consistent adherence to the Ministry of Health guidance on routine iron/folic acid supplementation among pregnant mothers in both settings. However, it is worth noting that the effect of iron/folic acid supplementation on haemoglobin levels in both settings could not be assessed in both settings due to a lack of equipment and supplies. Studies by Michael Habtu et al. [33], Sunita Taneja et al. [8], and Melesse Kuma et al. [34] revealed elevated haemoglobin levels among women in the intervention group, findings that our study was unable to replicate due to the constraints related to equipment and supplies.

The estimates showed no difference between the two settings for maternal episodes of headache, depression and diarrhoea across the various visits. This implies that these health concerns are common and not influenced by the study settings. These need to be addressed in both settings for the well-being of mothers.

Our investigation into the nutritional status of the infants revealed that integration of comprehensive NACS increased infant birth weights, and reduced instances of underweight, stunted and wasted infants. This implies that nutrition integration had a potential benefit of on foetal and infant growth and development. Our findings concur with; Veena et al. [35] M Barker et al. [9], Von Salmuth et al. [11], Olutayo et al. [12], Micheal Habtu et al. [36] on the effectiveness of a holistic approach to improving the nutritional status of children.

Routine NACS infants experienced significantly more episodes of diarrhoea and fever at the various visits than the comprehensive NACS group. The findings concur with Gonzalenz-Fernandez et al. [37] in their study in which implementation of the multisectoral approach lowered the risk of diarrhoea and respiratory infections. This implies that the health facilities in the routine NACS settings did not comprehensively address these health concerns hence the need for more interventions for better health and nutrition outcomes.

One of the strengths of this study lies in its comparison of two separate groups; routine versus comprehensive and its close monitoring of the practices and outcomes of the study participants. This approach bolstered the study’s findings, providing a clear and robust insight into the effectiveness of the integrated intervention package. Moreover, the study emphasizes favourable outcomes of comprehensive NACS highlighting the potential benefits of such comprehensive interventions, whose findings are also consistent with the existing literature. On the other hand, the study could not assess the impact of iron/folic acid supplementation on haemoglobin levels due to a lack of equipment and supplies, which is a limitation in understanding the complete maternal health and nutrition outcomes.

## Conclusions

The findings add to the existing body of evidence supporting improved maternal-infant health and nutrition practices and status with integrated nutrition services. While meal frequency, and iron/folic acid supplementation were similar in both groups, integration of comprehensive NACS intervention improved; maternal weights, infant birth weights, and infant growth in light of weight-for-age, length-for-age and weight-for-length. This emphasises the potential benefits of integrated nutrition interventions in promoting the overall being of the mothers and their infants.

## Recommendations

Based on the above findings, the Ministry of Health should consider: investing in acquiring the necessary equipment and supplies to assess the impact of iron/folic acid supplementation on haemoglobin levels for comprehensive evaluation of the women; scaling up integration of the comprehensive NACS in the health system as it has positive effect on the maternal-infant nutrition practices, health and nutrition outcomes; investing in digitization to ease monitoring and tracking trends in the health and nutrition status of the mother-infant pairs.

Future research can focus on the implementation and effectiveness of digitization in monitoring and tracking mother-infant health and nutritional status in an integrated health system. Secondly, it will be important to investigate the experiences of the women and caregivers receiving the nutrition services. In light of the research design, future research could focus on conducting cost-effectiveness and efficiency analysis to evaluate the economic implications of implementing comprehensive NACS programs compared to routine approaches. Additionally, impact evaluation studies on NACS integration in the health system could be conducted.

## Data Availability

The datasets generated and analysed during the current study are are available from the corresponding author on reasonable request.

## References

[1] Development Initiatives Poverty Research Ltd. Global Nutrition Report: stronger commitments for greater action. Bristol. UK. &view=article&id=472&Itemid=472; 2022. http://www.segeplan.gob.gt/2.0/index.php?option=com_content.

[2] UBOS. the Republic of Uganda Uganda Demographic and Health Survey (Udhs) 2022 Extension. 2023; September:6. accessed on 21st october 2023.

[3] Uganda Bureau of Statistics (UBOS) and ICF. Uganda Demographic and Health Survey 2016. 2016. www.DHSprogram.com.

[4] Marshall NE, Abrams B, Barbour LA, Catalano P, Christian P, Friedman JE (2022). The importance of nutrition in pregnancy and lactation: lifelong consequences. Am J Obstet Gynecol.

[5] Brink LR, Bender TM, Davies R, Luo H, Miketinas D, Shah N (2022). Optimizing maternal Nutrition: the importance of a tailored Approach. Curr Dev Nutr.

[6] Girard AW, Olude O. Nutrition education and counselling provided during pregnancy: Effects on maternal, neonatal and child health outcomes. Paediatr Perinat Epidemiol. 2012;26 SUPPL. 1:191–204. 10.1111/j.1365-3016.2012.01278.x 22742611

[7] Taneja S (2020). Impact of an integrated nutrition, health,water sanitation and hygiene, psychosocialcare and support intervention package delivered. Bmc.

[8] Taneja S, Upadhyay RP, Chowdhury R, Kurpad AV, Bhardwaj H, Kumar T (2021). Impact of nutritional interventions among lactating mothers on the growth of their infants in the first 6 months of life: a randomized controlled trial in Delhi, India. Am J Clin Nutr.

[9] Barker M, Dombrowski SU, Colbourn T, Fall CHD, Kriznik NM, Lawrence W, Norris SA, Ngaiza G, Patel D, Skordis-Worrall J, Sniehotta FF, Steegers-Theunissen R, Vogel C (2018). K Woods Townsend and JS. Intervention strategies to improve nutrition and health behaviours before conception. Lancet.

[10] Harriet, Torlesse. Nita Dalmiya VT and VA. Counselling to improve maternal nutrition: considerations for programming with quality, equity and scale. Unicef. 2021;:1–16.

[11] Von Salmuth V, Brennan E, Kerac M, McGrath M, Frison S, Lelijveld N (2021). Maternal-focused interventions to improve infant growth and nutritional status in lowmiddle income countries: a systematic review of reviews. PLoS ONE.

[12] Adeyemi O, Toure M, Covic N, van den Bold M, Nisbett N, Headey D (2022). Understanding drivers of stunting reduction in Nigeria from 2003 to 2018: a regression analysis. Food Secur.

[13] Akseer N, Vaivada T, Rothschild O, Ho K, Bhutta ZA (2020). Understanding multifactorial drivers of child stunting reduction in exemplar countries: a mixed-methods approach. Am J Clin Nutr.

[14] Bhutta ZA, Akseer N, Keats EC, Vaivada T, Baker S, Horton SE (2020). How countries can reduce child stunting at scale: lessons from exemplar countries. Am J Clin Nutr.

[15] 2020; USAID Advancing Nutrition. Stunting: considerations for use as an indicator in nutrition projects. Arlington, VA: USAID Advancing Nutrition, September. https://www.advancingnutrition.org/sites/default/files/2021-10/usaid_an_stunting_literature_review_2021.pdf.

[16] Dearden KA, Bishwakarma R, Crookston BT, Masau BT, Mulokozi GI (2021). Health facility-based counselling and community outreach are associated with maternal dietary practices in a cross-sectional study from Tanzania. BMC Nutr.

[17] Kaleem R, Adnan M, Nasir M, Rahat T (2020). Effects of antenatal nutrition counselling on dietary practices and nutritional status of pregnant women: a quasi-experimental hospital based study. Pakistan J Med Sci.

[18] Pérez-Escamilla R, Segura-Pérez S, Hall Moran V (2019). Dietary guidelines for children under 2 years of age in the context of nurturing care. Matern Child Nutr.

[19] Ghosh-Jerath S, Devasenapathy N, Singh A, Shankar A, Zodpey S. Ante natal care (ANC) utilization, dietary practices and nutritional outcomes in pregnant and recently delivered women in urban slums of Delhi, India: an exploratory cross-sectional study. Reprod Health. 2015;12. 10.1186/s12978-015-0008-9 PMC4396888 25889714

[20] Nsiah-Asamoah C, Pereko KKA, Intiful FD (2019). Nutritional counselling interactions between health workers and caregivers of children under two years: observations at selected child welfare clinics in Ghana. BMC Health Serv Res.

[21] MINISTRY OF HEALTH. Guidelines on Maternal, Infant, Young Child and Adolescent Nutrition. Ministry of Health, Uganda. 2021. https://www.health.go.ug/cause/guidelines-on-maternal-infant-young-child-and-adolescent-nutrition/.

[22] World Health Organization (2020). WHO recommendations on antenatal care for a positive pregnancy experience.

[23] Ministry of Health U. INTEGRATING NUTRITION ASSESSMENT. COUNSELLING, AND SUPPORT INTO Training Course for Facility-Based Health Providers. 2016.

[24] Levin CE, Self JL, Kedera E, Wamalwa M, Hu J, Grant F (2019). What is the cost of integration? Evidence from an integrated health and agriculture project to improve nutrition outcomes in Western Kenya. Health Policy Plan.

[25] Kasiulevičius V, Šapoka V, Filipavičiūtė R (2006). Sample size calculation in epidemiological studies. Gerontologija.

[26] Beal SJ, Kupzyk KA (2014). An introduction to Propensity scores: what, when, and how. J Early Adolesc.

[27] Valojerdi AE, Janani L (2018). A brief guide to propensity score analysis. Med J Islam Repub Iran.

[28] Garrido MM, Kelley AS, Paris J, Roza K, Meier DE, Morrison RS (2014). Methods for constructing and assessing propensity scores. Health Serv Res.

[29] Dehejia RH, Wahba S (2002). Propensity score-matching methods for nonexperimental causal studies. Rev Econ Stat.

[30] Taylor RM, Wolfson JA, Lavelle F, Dean M, Frawley J, Hutchesson MJ (2021). Impact of preconception, pregnancy, and postpartum culinary nutrition education interventions: a systematic review. Nutr Rev.

[31] Kim C, Mansoor GF, Paya PM, Ludin MH, Ahrar MJ, Mashal MO et al. Review of policies, data, and interventions to improve maternal nutrition in Afghanistan. Matern Child Nutr. 2020;16. 10.1111/mcn.13003 PMC7507462 32293806

[32] Tsegaye D, Tamiru D, Belachew T. Theory-based nutrition education intervention through male involvement improves the dietary diversity practice and nutritional status of pregnant women in rural Illu Aba Bor Zone, Southwest Ethiopia: a quasi-experimental study. Matern Child Nutr. 2022;18. 10.1111/mcn.13350 PMC9218320 35315583

[33] Habtu M, Agena AG, Umugwaneza M, Mochama M, Munyanshongore C. Effect of integrated nutrition-sensitive and nutrition-specific intervention package on maternal malnutrition among pregnant women in Rwanda. Matern Child Nutr. 2022;18. 10.1111/mcn.13367 PMC9218321 35538044

[34] Kuma MN, Tamiru D, Tefera B (2023). Effects of nutrition education and home gardening interventions on feto-maternal outcomes among pregnant women in Jimma Zone, Soutwest Ethiopia: a cluster randomized control trial. Nutr Health.

[35] Singh V, Ahmed S, Dreyfuss ML, Kiran U, Chaudhery DN, Srivastava VK (2017). An integrated nutrition and health program package on IYCN improves breastfeeding but not complementary feeding and nutritional status in rural northern India: a quasi-experimental randomized longitudinal study. PLoS ONE.

[36] Habtu M, Agena AG, Umugwaneza M, Mochama M, Munyanshongore C. Effectiveness of Integrated Maternal Nutrition Intervention Package on Birth Weight in Rwanda. Front Nutr. 2022;9 July. 10.3389/fnut.2022.874714 PMC9353189 35938121

[37] González-Fernández D, Mazzini Salom AS, Herrera Bendezu F, Huamán S, Rojas Hernández B, Pevec I et al. A multi-sectoral Approach improves early child development in a Disadvantaged Community in Peru: role of Community Gardens, Nutrition Workshops and enhanced Caregiver-Child Interaction: Project Wawa Illari. Front Public Heal. 2020;8 November. 10.3389/fpubh.2020.567900 PMC7681241 33240834

